# Effectiveness of olive oil for the prevention of pressure ulcers caused in immobilized patients within the scope of primary health care: study protocol for a randomized controlled trial

**DOI:** 10.1186/1745-6215-14-348

**Published:** 2013-10-23

**Authors:** Inmaculada Lupiáñez-Pérez, Juan Carlos Morilla-Herrera, Leovigildo Ginel-Mendoza, Francisco Javier Martín-Santos, Francisco Javier Navarro-Moya, Rafaela Pilar Sepúlveda-Guerra, Rosa Vázquez-Cerdeiros, Magdalena Cuevas-Fernández-Gallego, Isabel María Benítez-Serrano, Yolanda Lupiáñez-Pérez, José Miguel Morales-Asencio

**Affiliations:** 1 District of Primary Health Care Malaga-Guadalhorce, Andalusian Health Service, Malaga, Spain; 2 District of Primary Health Care Costa of Sol, Andalusian Health Service, Malaga, Spain; 3 University Hospital Virgen de la Victoria, Malaga, Spain; 4 Regional Hospital Carlos Haya, Malaga, Spain; 5 Faculty of Health Sciences, University of Malaga, Malaga, Spain

**Keywords:** Hyperoxygenated fatty acids, Olive oil, Pressure ulcers, Prevention

## Abstract

**Background:**

Pressure ulcers are considered an important issue, mainly affecting immobilized older patients. These pressure ulcers increase the care burden for the professional health service staff as well as pharmaceutical expenditure. There are a number of studies on the effectiveness of different products used for the prevention of pressure ulcers; however, most of these studies were carried out at a hospital level, basically using hyperoxygenated fatty acids (HOFA). There are no studies focused specifically on the use of olive-oil-based products and therefore this research is intended to find the most cost-effective treatment and achieve an alternative treatment.

**Methods/design:**

The main objective is to assess the effectiveness of olive oil, comparing it with HOFA, to treat immobilized patients at home who are at risk of pressure ulcers. As a secondary objective, the cost-effectiveness balance of this new application with regard to the HOFA will be assessed. The study is designed as a noninferiority, triple-blinded, parallel, multi-center, randomized clinical trial. The scope of the study is the population attending primary health centers in Andalucía (Spain) in the regional areas of Malaga, Granada, Seville, and Cadiz. Immobilized patients at risk of pressure ulcers will be targeted. The target group will be treated by application of an olive-oil-based formula whereas the control group will be treated by application of HOFA to the control group. The follow-up period will be 16 weeks. The main variable will be the presence of pressure ulcers in the patient. Secondary variables include sociodemographic and clinical information, caregiver information, and whether technical support exists. Statistical analysis will include the Kolmogorov-Smirnov test, symmetry and kurtosis analysis, bivariate analysis using the Student’s t and chi-squared tests as well as the Wilcoxon and the Man-Whitney U tests, ANOVA and multivariate logistic regression analysis.

**Discussion:**

The regular use of olive-oil-based formulas should be effective in preventing pressure ulcers in immobilized patients, thus leading to a more cost-effective product and an alternative treatment.

**Trial registration:**

Clinicaltrials.gov identifier: NCT01595347.

## Background

Pressure ulcers are considered to be an economic, social and health problem that does not just decrease the quality of life of the patients and their social and familiar environment but also involves the worsening of the patients’ prognosis, as well as decreasing the patients’ life expectancy, owing to the high number of related physical complications. Pressure ulcers may be defined as injuries to the skin caused by the ischaemic process that may affect and necrotize those areas in the epidermis, dermis, subcutaneous tissues, and muscles where the pressure ulcers appear, and may also affect bones and joints in the most severe cases. Pressure ulcers tend to appear when the soft tissues are compressed between two layers, namely, the bony prominences of the patient and an external surface [1]. Additionally contributing to their appearance are the vascular occlusion produced by the external pressure and the endothelial damage to arterioles and microcirculation, mainly due to tangential shear and frictional forces. Pressure ulcers involve the alteration of a basic need for patients, which is to preserve skin integrity. Pressure ulcers may appear in any part of the body, with the bony prominences (sacrum, hips, heels) being most common, and mainly affect older people, immobilized patients with severe acute disease and patients with neurological deficiencies.

The incidence and prevalence of pressure ulcers are some of the most representative indicators of the quality of nursing care. The data on pressure ulcer prevalence in Spanish hospitals (8.24%) [2] are very similar to its neighboring countries, Italy (8.3%), France (8.9%), Germany (10.2%), and Portugal (12.5%) [3,4] or countries further afield, such as Jordan (12%) [5]. The highest records of prevalence are registered in Ireland (18.5%), Wales (26.7%) [6], Belgium (21.1%), the United Kingdom (21.9%), Denmark (22.7%), and Sweden (23.0%) [7]. The appearance of pressure ulcers in hospitalized patients is a frequent complication, which has a negative impact on the patient’s health and often causes an extension of the hospitalization period and, thus, an increase in healthcare expenses.

With regard to nursing care at home, there are no specific records that compare the prevalence in patients included in programs of nursing care at home given that the characteristics of the health systems vary considerably from one country to another. In nursing homes, the most important study carried out might be the one recently published by Park-Lee [8], which analyzes a sample of American nursing homes for older people and reports a pressure ulcer prevalence of (11%), similar to the Spanish rate.

With regard to primary healthcare, there are no specific data concerning patients who receive nursing care at home with which to make a comparison on the prevalence of pressure ulcers in Spain with different countries, given that the characteristics of the health systems vary substantially from one country to another. According to data obtained from the second National Study on Pressure Ulcers Prevalence in Spain, in 2005 [9], the crude prevalence pressure ulcers was registered at 3.73% and the mean prevalence rate was registered at 9.11% ± 10.9% for those patients over 14 years who are included in the nursing care at home service. The most common ulcer sites are the sacrum and heels, affecting mainly the population group, including people over 65 years. The Third National Study on Pressure Ulcers Prevalence in Spain shows that in 2009 [2] the crude prevalence was 5.89%. This rising percentage is due to the Spanish population aging, since this study was carried in 2005 up to 2009.

Today, pressure ulcers are considered an important health problem involving a financial impact, which substantially increases pharmaceutical expenditure. The total spending derived from the treatment of pressure ulcers represents 5% of the annual healthcare spending [10], and generates a heavier health care workload for the professional staff.

The main risk factors causing pressure ulcers are immobility, incontinence, malnutrition, and awareness level. Pressure ulcers are an indirect indicator of health care quality, thus a low prevalence of pressure ulcers in patients suggests a higher quality in the health care services, based on the use of preventative measures. The assessment of risk factors, the use of support surfaces, the repositioning of the patient, a good nutritional status, and skin moisturizing or the use of checklists are adequate strategies to prevent pressure ulcers [11-18] that have been proved effective even in older patients [19].

A number of products are intended for the prevention of pressure ulcers, such as oil compounds based on hyperoxygenated fatty acids (HOFA). A large number of studies have proven their effectiveness in avoiding pressure ulcers or by postponing the appearance of such pressure ulcers by maintaining skin integrity [20-25]. However, one inconvenience of this treatment is its high cost in the long term.

In Mediterranean cultures, olive oil is an essential element with proven effects as a basic component of the diet in the prevention of cardiovascular disease. However, together with its nutritional properties, it also provides beneficial effects when applied topically, mainly owing to its moisturizing and emollient qualities, and its composition has led to its use as an ointment. Olive oil is composed of 98% triglycerides, including predominantly monounsaturated oleic acids, which owing to their antiinflammatory properties have been proven to be essential for health and skin maintenance, as such properties are similar to ibuprofen (recent studies attribute this to Oleocanthal), and this may accelerate the recovery and healing process of wounds [26]. Furthermore, owing to the high concentration of polyphenols, which are natural antioxidants, included in extra virgin olive oil, its use mitigates the inflammatory process, thus explaining its beneficial effects for skin inflammatory disorders [27,28]. The role of oleic acid is a key feature within the reconstruction of cell membranes, providing higher smoothness to the dermis by restoring skin humidity levels, thus moisturizing the skin and providing it with elasticity. Besides, such oil components as phenolic compounds and chlorophyll have a high antioxidant effect and are therefore anti-aging, apart from accelerating the dermis healing process. Moreover, it should also be mentioned that vitamin E is included in the oil composition, which is an excellent source of protection against the free radicals causing cell oxidation [29]. An excess of free radicals in the organism accelerates aging and as a result, epithelial cell membranes are modified, thus making skin nutrition difficult, and also damaging the collagen and elastin fibres, so that the skin loses its firmness and elasticity. These free radicals include a 7-electron oxygen atom: the stable oxygen atom has eight electrons and becomes unstable when it loses one electron. When such an electron is missing, it is borrowed from the cell membrane and produces another free radical, causing a chain reaction. This is counteracted by the action of antioxidants, which neutralize the oxygen atoms. The exogenous antioxidants acting in the membrane lipids include vitamin E, carotene, polyphenols, and flavonoids, which makes extra virgin olive oil a natural source of such components [30].

The main role of the oil is its antioxidant function: for this purpose, the oil should have a low peroxide content and a high polyphenol content. Extra virgin olive oil includes 330 to 500 mg of polyphenols per kg of fat and less than 20 milliequivalent (mEq) weight of peroxide per kg of fat (the composition of the HOFAs includes from 40 to 50 mg of polyphenols per kg of fat and from 230 to 340 mEq of peroxide per kg of fat). Owing to its lipid composition, it is completely compatible with of human tissue cells, which means that its topical use does not cause allergy or irritation. Such properties lead us to consider a possible protective effect on the appearance of pressure ulcers, similar to those of HOFA, but at a much cheaper cost, as well as offering an alternative treatment option. Furthermore, there are no previous experiments with this product in the prevention of pressure ulcers in nursing care at home. The main aim of this trial is to assess whether the effectiveness of olive oil use is lower or higher than the HOFA in the prevention of pressure ulcers in immobilized patients receiving nursing care at home and, as a secondary aim, to assess the cost-effectiveness of this new intervention with regard to the use of HOFA.

## Methods/design

The design of this study is a noninferiority, triple-blinded, parallel, multi-center, randomized clinical trial. The blinded groups are the patients and their caregivers, nurses and the research group.

The null hypothesis of this study is that the difference in the appearance of pressure ulcers in immobilized patients at home who receive olive oil (*G*_o_), in contrast to those patients applying HOFA (*G*_h_) is higher than the delta value established at 10% (*H*_0_ = *G*_o_ − *G*_h_ > 10%), that is to say, that the lower bound of the confidence intervals includes that 10%. In the event that it is possible to reject the null hypothesis, the alternative hypothesis of this study will be that olive oil is not lower than 10%, compared with the HOFA (*H*_1_ = *G*_o_ − *G*_h_ < 10%); in other words, the lower bound of the confidence interval of the difference between both products is lower than 10%.

Two procedures will be performed: usual care and application of HOFA to the control group and usual care and application of an olive-oil composition to the target group. The main outcome will be the appearance of grade 2 pressure ulcers. The accuracy of the trial with regard to the control medicine (HOFA) is widely supported, owing to the favorable historical results of such medicine against placebo [25] as per recommendations of the International Conference of Harmonization and also because the efficiency conditions of the mentioned medicine will be met for the purpose of this trial.

The target population will be patients included in the immobilized-patients program within the nursing care at home service provided in Health Centers in Andalucía (Spain), and more specifically in the regions of Malaga, Granada, Seville, and Cadiz.

The inclusion criteria will be: patients over 18 years; not having pressure ulcers; patients supported by a family or a remunerated caregiver for the application of the treatment; diagnosed by a nurse as a ‘risk for impaired skin integrity’, by applying the Braden Scale for prediction of pressure ulcer risk, showing a high risk (score ≤ 12) or moderated risk (score 13 to 16), and; patients with nutritional status assessed according to the Mini Nutritional Assessment scale scoring 10 or less.

As exclusion criteria, the following were established: the patient declines to take part in the trial; patients with a usual address different from the area of the health center where the study is carried out, or plans to be away from the usual address during the follow-up period; those patients who have been hospitalized during the sampling period; terminally ill patients or those who already have pressure ulcers.

All the patients included in the trial will receive the information concerning the aim of the trial in writing, which will be duly signed by the researchers. The patients will confirm their participation in the trial and sign an informed consent form. In those cases when the patients have cognitive impairment, the legal guardian will be the one who signs the informed consent form. Patients who do not confirm their consent will be substituted by other patients selected by the same procedure of general randomized selection, thus, extending the selection process as long as necessary.

### Sample selection

The directors and care service coordinators of each health center will be duly informed about the trial, and they will be asked for their availability as well as their consent to take part in the trial. Nursing staff of each health center who so desire will be able to take part, being in such case responsible for the research study implementation and follow-up in their respective health center.

Records of patients included in the nursing at home program for immobilized patients will be assessed to determine whether patients comply with the inclusion requirements. At this point, such patients will sign the informed consent form and subsequently, their details will be randomized by a computer system independent of the professional staff, which will be processed at the clinical trial control center.

To achieve a power rate of 80.00% to reject the null hypothesis (*H*_0_: the difference between proportions *p*1 and *p*2 is lower than the noninferiority bound), using a normal asymptotic analysis test (noninferiority), unilateral proportions for two independent samples, taking into account that the level of statistical significance is 5.00%, and assuming that the proportion in the reference (control) group is 45.00% and the proportion in the experimental group is 45.00% [9], the proportion of experimental units in the reference group is 50% related to the total and the noninferiority bound (delta) is 10.00%, then, it will be necessary to include 306 experimental units in the reference group and 306 units in the experimental group, totalling 612 units in the study. Taking into account that the expected withdrawal rate is 15.00%, it will be necessary to enroll 360 experimental units in the reference group and 360 units in the experimental group, totalling 720 experimental units in the trial.

The main outcome will be the incidence of grade 2 pressure ulcers in patients during the 16-weeks of follow-up (112 days). We have selected this grade because when the patient has grade 2 pressure ulcers, prevention can no longer be carried out, and prevention is the intended outcome. We are only able to conduct this trial on intact skin, therefore it is only possible to conduct the trial when the patients have no pressure ulcer or have grade 1 pressure ulcers, when there are just erythemas. Therefore, patients who develop grade 2 pressure ulcers with breaks in the skin are excluded from the trial, as we cannot keep using the products because prevention is not useful anymore. Such patients will be treated again by the nurse who will cure the pressure ulcer, although this will be done out of the trial. When preventing the patient from developing grade 2 pressure ulcers, we are also preventing greater stages of ulcer. The 112 days of follow-up are higher than trials carried out up to now, 7 days [21], 9 days [24], and 30 days [25], which is more than enough for the appearance of pressure ulcers at any stage. The appearance of grade 2 pressure ulcers will be confirmed by the inspection of those areas where the product has been applied (sacrum, hips, and heels).

As secondary endpoints, cost measures will be applied that will allow us to assess the cost-effectiveness balance of the new product (olive-oil formula) against the HOFA-based products. As inputs of this model, the cost of each treatment will be used and defined as the number of units (NE_*ij*_) necessary to carry out 16 weeks’ treatment (112 days) multiplied by the price of one unit (*P*_*i*_ and *P*_*j*_) (the price of each unit will be achieved from the information provided by each supplier according to the market price). The number of units will be the result of dividing the treatment period (DT_*ij*_) (112 days) by the number of days (*d*_*ij*_) the recipient used, according to the dose established in the trial protocol in each branch of the treatment. The resulting amount achieved shall be rounded up, even if the whole number of units has not been fully used:

$${\mathrm{NE}}_{\mathrm{ij}}={\mathrm{DT}}_{\mathrm{ij}}/d_{\mathrm{ij}}$$

As a variable cost, the number of visits of the nurse to the patient’s home during the trial period (VE_*i*_) will be used, which will act as a proxy measure of the level of care required. The mean time spent on each visit will be estimated according to the standard value of time within the community-health nurse working hours spent during a nursing care at home visit (this information will be obtained from the human resources management divisions of the different health districts and an assessment test will be carried out on the length of 30 visits to patients with the characteristics complying with the inclusion criteria, randomly selected among the health centers included in the study, prior to the commencement of the trial). Structural fixed costs will not be included, as in all cases the care assistance is provided at the patient’s home and the services are already included in the aforementioned variable. It will not be possible to assess the cost derived from the family care, since this is quite difficult to achieve and has a high variability. Therefore, this will not be included in the model. In those cases when there is a remunerated caregiver, such cost will not be included in the total costs either, as it would create a bias in the estimation when comparing them with those patients who are not assisted by a remunerated caregiver. Furthermore, the intangible costs derived from certain aspects, such as the pain level derived from the process will not be included either. Therefore, the estimation of total costs (CT) in the model will remain as follows:

$${\mathrm{CT}}_{\mathrm{ij}}={\mathrm{NE}}_{\mathrm{ij}}+{\mathrm{VE}}_{\mathrm{ij}}{}_{.}$$

The effectiveness variable will be the appearance of pressure ulcers. A limited number of states will be taken into account: appearance of pressure ulcers (*U*_1_) and nonappearance of pressure ulcers (*U*_0_). The incidence will be estimated in percentages, which will be used for the incremental estimations on cost-effectiveness.

Furthermore, variables on characterization of patients and caregivers will be included to assess sociodemographic data, such as the time registered in the care service for immobilized patients, information about previous cases of pressure ulcers, comorbidity, nutritional and cognitive status, and dampness (incontinence), apart from taking into account the ulcer location as well as the availability of technical support items (mattress and cushions to avoid bedsores, adjustable beds, and so on). With regard to the caretaker, the age, sex and whether he or she is a family or remunerated caregiver will be taken into account.

A basal-assessment will be performed on all patients at the beginning of the trial and this will be repeated each week up to the conclusion of the follow-up period, or until a pressure ulcer appears in patients. All the information concerning the application of the products will be registered in the weekly evaluations.

#### Procedure

The new procedure consists of the application of a formula, in either liquid or spray, which contains 95% extra virgin olive oil. This product will have the same appearance as the hyperoxygenated fatty acid, using the same presentation, which will make it appear identical to both patients and nurses. This intervention will be applied to both groups (control and target group). Both the patients of the control group and those patients who are part of the target group will receive the preventative instructions stated in the clinical practice guidelines on deterioration of skin integrity issued by the Health District of Malaga. For such purposes, the caregivers of both groups will be trained in the mentioned procedures. In addition to these preventative measures, the patients included in the control group will receive two applications per day of the HOFA-based product in the skin, more specifically, in the sacral area, the hips, and the heels. For the purpose of this trial, we will use a HOFA product bearing the CE marking, IIb Class, with a higher classification than other products existing in the market. The product is to be applied topically and includes hyperoxygenated fatty acids, *Equisetum Arvense*, *Hypericum Perforatum*, and perfume. On their part, the target group will receive, as well as the preventative measures mentioned above, two daily applications of the olive-oil-based formula in the skin areas of sacrum, hips, and heels. The appearance of pressure ulcers will be registered in the weekly follow-up report, which will include an assessment of whether the skin integrity is maintained or not. In the event that there is any adverse effect during the follow-up period, the research group will be informed and such events will be notified by means of a specific document designed for such purposes.

#### Statistical analysis

Initially, a descriptive analysis of the variables included in the trial will be performed. Continuous variables will be summarized with mean, standard deviation, median, and interquartile range. Categorical variables will be shown in absolute and relative frequencies. An analysis of the normal distribution by the Kolmogorov-Smirnov test, symmetry analysis, and kurtosis will be performed.

With the aim of assessing the noninferiority of the olive oil for the prevention of pressure ulcers, a contrast of association between the appearance of pressure ulcers and the kind of treatment received (olive-oil-based cream or conventional treatment) will be carried out, by absolute and relative measures of effect sizes (complete reduction of risk, number needed to treat, relative risk, and relative reduction of risk), with their respective confidence intervals at 95%. It will be determined whether the confidence interval includes the estimated delta value from the sample or not, so as to reject or accept the null hypothesis.

A bivariate analysis will be carried out using the Student’s t and chi-squared tests according to the characteristics of the variables analyzed in the event that these are normally distributed. Otherwise, nonparametric tests will be applied, such as the Wilcoxon and Man-Whitney U tests. Likewise, analysis of variance (ANOVA) will be applied for the association of quantitative and qualitative variables in the necessary cases, as well as robust measures of central tendency in cases of homoscedasticity (which will be tested by Levene’s test) by means of the Welch and Brown-Forsythe test.

An analysis according to the Kaplan-Meier curves and log-rank test will be carried out to determine the progress of the appearance of pressure ulcers in both groups, taking into account the treatment applied, the sex, age, nutritional status, and functional degree of the patient and the caregiver’s age.

For the analysis on cost-effectiveness, the effectiveness measures previously established (*U*_1_ and *U*_0_) will be obtained. The increase in cost for each option (Δ*C*_*ij*_) will be estimated, as well as the increase of effectiveness rate, when comparing the two options, the cost-efficiency ratio of each treatment and the incremental cost-effectiveness ratio of each option. These values will enable us to assess the necessary cost to increase by 1% the possibilities of success when preventing pressure ulcers in these patients by using each product.

The analysis will be carried out according to the protocol, as established for the noninferiority studies, to ensure the difference conditions between the treatments and increase the rejection conditions of the null hypothesis. Nevertheless, an analysis will be carried out that is intended to compare both analyses and assess, if the analyses differ, the subgroups of patients who did not comply with the trial protocol with the aim of identifying the possible reasons for the withdrawal from the trial, before accepting or rejecting the null hypothesis. The confidence intervals of the main result variable will be analyzed to determine whether the difference found is within the bound established for the delta value. Statistical analysis will be performed using SSPS 20 software.

#### Ethical and legal issues

The Ethics and Research Committee of Malaga (Northwest District) granted the approval for this trial. The regulations on Good Clinical Practice will be observed at all times, as well as the ethical principles established for the research on human beings stipulated in the Declaration of Helsinki and further amendments thereto. The clinical information will remain separate from the identification details and the databases will be coded and stored in specific computers solely intended for the project. All the records will be made observing all the dispositions from the legislation in force with regard to personal data protection according to the Act 15/1999 dated 13 December, as well as on safety of computerized files which may store personal data information, and more specifically, with regard to the access through communication networks (Royal Decree 994/1999 dated 11 June) and access to confidential information for scientific purposes from the Commission Regulation CE No. 831/2002 by the European Union and Act 41/2002 dated 14 November, which is considered the legal standard on Patient’s Autonomy and Rights and Duties in terms of Clinical Information and Documentation.

The staff responsible for such purpose will manage the data according to the instructions provided by the person responsible for the treatment. Such data will not be applied or used for a different purpose than those stated in the respective authorization; neither will they be reproduced to other people even for registration purposes. Once the aim of the trial has been carried out, the personal data collected will be destroyed, deleted, or given back to the person responsible for the treatment, along with any other media or documents containing any personal data related to the project. Every patient will be informed verbally and in writing about the objectives of the project and its methodology. Each individual will sign the respective informed consent form.

## Discussion

Pressure ulcers are nowadays considered an important health issue causing a great economic impact by substantially increasing pharmaceutical expenditure. The total expenditure of pressure ulcer treatments amounts to 5% of annual health care expenditure and causes a higher health care burden on professional staff. This is a public health problem, which affects mainly older people, thus, this disease rate is increasing.

The acceptance of our hypothesis will provide evidence supporting the regular use of olive oil (nonoxygenated fatty acids) in the prevention of pressure ulcers in primary health care, likewise involving hospital care. Furthermore, owing to the lower price of the product, primary health care districts may consider purchasing them, owing to the reduction in health care workload for professional staff, as well as for the family caregivers, given that the incidence of pressure ulcers will be lower as well as the respective reduction in pharmaceutical expenditure.

The main limitation of the trial is that when performing the cost-effectiveness analysis, the variables on caregiver inputs may not be estimated, along with those variables regarding the expenditure derived from the patient’s suffering. Likewise, there might be a selection bias due to those patients who refuse to take part in the trial, which may involve differential baseline characteristics. For the purposes of this trial, in the analysis it will be taken into account whether there are differences among those who take part in the trial and those who do not. The same procedure will be applied for withdrawals during the follow-up period. The analysis will be carried out according to the protocol and intention to treat. In the event that the results of the two types of analysis do not match, the subgroup of patients who did not comply with the protocol will be studied, to determine the reasons before reaching a final conclusion of noninferiority.

The confusion bias derived from some variables such as age, sex, type of intervention, and nutritional status of the patient will be managed during the statistical analysis stage, by means of a multivariate analysis.

The usual application of olive-oil-based formulas may be helpful in preventing the appearance of any grade of pressure ulcer in immobilized patients, and thus, it will be a cheaper treatment and may be considered as an alternative treatment.

## Trial status

The randomized trial is currently in the phase of patient’s enrolment and follow-up.

## Abbreviations

ANOVA: Analysis of variance; HOFA: Hyperoxygenated fatty acids; mEq: Milliequivalent.

## Competing interests

The authors declare that they have no competing interests.

## Authors’ contributions

ILP has taken part in the original conception and design of this study and drafted the first version of this project as well as the manuscript. JMMA, JCMH, LGM, FJMS, FJNM, RPSG, RVC, MCFG, IMBS, and YLP have taken part in the conception and design of the study and critically reviewed the draft of the manuscript, providing a key intellectual contribution to the final version. All authors have read and approved the final manuscript.
